# Temporal trend of the first prescription of nevirapine: the ANRS CO3 Aquitaine Cohort, 1997-2008

**DOI:** 10.1186/1758-2652-13-S4-P37

**Published:** 2010-11-08

**Authors:** M Bruyand, S Geffard, S Lawson-Ayayi, F Dauchy, G Miremont-Salamé, C Greib, S Farbos, P Morlat, F Dabis

**Affiliations:** 1 INSERM U897, 146 rue Léo saignat, Bordeaux, France; 2 Services de médecine interne et maladies infectieuses, CHU de Bordeaux, Bordeaux, France; 3 INSERM U657, Bordeaux, France; 4 Service de médecine, CHR de Bayonne, Bayonne, France; 5 Services de médecine interne et maladies infectieuses, Hôpital Saint André (CHU), Bordeaux, France

## Purpose

Nevirapine (NVP) is commonly prescribed in antiretroviral therapy (ART). We aimed at describing the evolving characteristics of the patients receiving a first prescription of NVP over 12 years in the ANRS CO3 Aquitaine Cohort, a French hospital-based HIV1-infected cohort.

## Methods

All HIV1-positive patients of the participating clinic wards, aged over 13 and giving informed consent are included in the cohort. Patients receiving a first prescription of NVP between 1997 and 2008 are described at baseline and during follow-up according to their treatment status, ART-naïve or not. Chi-square test, signed rank Wilcoxon test, Mc Nemar test and log-rank test are used for comparisons.

## Results

Among 5,566 cohort participants, 1,775 received a first NVP-based regimen during the study period, and 277 (16%) of them were naïve of ART at the time of NVP introduction. Pre-treated patients received ART prior to NVP for a median duration of 47 months (IQR: 27-76). The ratio pre-treated : naïve patients increased from 4.7:1 in 1997-1999 to 14.5:1 in 2006-2008, whereas, respectively 476 and 47 patients on average initiated NVP each year in these periods.

At the time of NVP initiation, the median age of the ART-naïve group was 36 years, vs 39 years in the pre-treated one (p<0.001). Women accounted for 29% of both groups. The naïve patients were rarely at the AIDS stage 4.7% vs 23.4% in the pre-treated group (p<0.001). The median CD4 cell counts in the naïve and pre-treated groups were 365 and 390 cells/mm^3^, respectively (p=0.32), and the median plasma HIV RNA loads were 19,000 and 2,200 copies/mL in naïve and pre-treated patients, respectively (p<0.001).

Pre-treated patients were more likely to interrupt the NVP-based treatment (p<0.001). Within 6 months after NVP initiation, 61 (22%) ART-naïve patients and 394 (26%) pre-treated patients interrupted NVP. After one year these proportions were 32% and 41%, respectively. Clinical or viro-immunological failure represented 40% of the causes of NVP interruption in pre-treated patients and 18% in naïve patients. Drug toxicity represented respectively 21% and 25% of the causes of NVP interruption in these groups.

## Conclusion

The number of annual initiation of NVP based ART has decreased in the Aquitaine Cohort until 2005, and is stable since then. After one year of treatment, 40 % of the patients had interrupted the NVP-based regimen. The main causes of discontinuation were clinical or viro-immunological failure and drug toxicity.

